# Extra Supplement.-The Nursing Mirror

**Published:** 1888-10-06

**Authors:** 


### The Nursing Mirror.

All Contributions for this Supplement should be addressed "The Nursing Mirror," care of The Editor, 38, Old Jewry, E.C.

### En Passant.

A DOLL SHOW.—Many of our readers must have seen in the daily papers and elsewhere the announcement that a doll show is to be held at the Hospital for Sick Children, Great Ormond Street, on November 5th. There will be three prizes of five, three, and one pounds given for the best-dressed and most effective dolls, and after the prizes have been awarded the dolls will be sold for the benefit of the hospital. The dolls must be sent in by November 1st, together with an entrance fee of one shilling for each doll, and they must all be plainly labelled with the competitor's name and address. This delightful scheme, by which the energetic Lady Superintendent of Great Ormond Street hopes to raise funds, will be greatly forwarded if nurses will compete by dressing dolls in different hospital uniforms. It is certain that, at a sale held in a hospital, a doll dressed as a nurse or sister will be readily sold; the costume is effective also, and should attract the judges. The dolls may be of any size, for the prizes will be given for the dressing and general effect, so that those who cannot afford to buy an expensive doll will stand as good a chance as their richer rivals. A concert is to be given before the show, at which some of our best singers have promised to perform. The Princess Frederica will present the prizes.

NURSES AND UNIFORM.—Amongst the other improvements which are being inaugurated at the Children's Hospital, Great Ormond Street, is the adoption of a new uniform. Probationers, lady pupils, and staff nurses are now all dressed in pink gowns, and, though the staff nurses wear a slightly different cap, there is no distinction whatever between the two sets of probationers. The sisters have claret-coloured gowns, which are becoming and novel in colour, but their caps and aprons are rather plain: if the aprons had straps or the caps had strings, the uniform would gain in its power of conferring dignity. The new uniforms were worn for the first time at the Harvest Festival, held last Saturday in the exquisite little chapel within the hospital. The wards were thrown open to visitors, and looked their very best, and the chapel was beautifully decorated, and the service well conducted. It must be a great pleasure to the new Lady Superintendent to be able to keep on all the old nurses, for they have been trained to take entire charge of both chapel and mortuary. As a rule, there is no more ghastly place on earth than a hospital mortuary, but the exception is to be found at Great Ormond Street. Decorated like a chapel, lighted with gas, and with a small altar bearing a cross at one end, it is a fitting place for the poor dead babes to await burial. A record is kept of all the little ones who have lain within its walls, and a careful selection of texts are written up for the comfort of those who come here to claim their dead. Surely, at all children's hospitals, the nurses might have charge of the mortuary, and keep it with the exquisite care and thought shown at Great Ormond Street.

MASSAGE.—We receive many questions about massage, and are afraid our correspondents are often disappointed at our answers, but it is essentially an operation to be seen, not read about. Still, to do our best to please our readers, we give the following directions from a clinical lecture by Dr. Hale White, which was published in the *British Medical Journal:* "First grease the parts with vaseline or oil, and if the skin be very hairy it may be necessary to cut the hair; then stroke the muscles firmly several times with the edge of the hands in the direction of the venous flow. In places, such as the back, where this is impossible, always stroke in the same direction; on the abdomen, follow the course of the colon; next you may take up the skin between the thumb and forefinger, and rub it between them, one hand following the other, in the same direction as the stroking, then thoroughly knead the muscles with one or both hands, according to the size of the part, in the same direction as already mentioned; after this, move all the joints in every direction, then you may conclude by striking the muscles with several small blows—but this is not of much importance. The full time should be about an hour twice a-day for the whole body. If there is a painful spot and the case be one of hysteria, particularly direct your energies to that part; of course, avoid the bones."

NURSES AND COURAGE.—Our remarks on the necessary courage required for nursing infectious fevers seem likely to develop into a discussion on vaccination, which is quite beyond our province. A physician sends us a leaflet entitled "The Vaccination Question," by Dr. George Cordwent, of Milverton, which gives in a clear, concise form the objections to vaccination, but none of the reasons in its favour. With regard to the nursing of typhus, the tragic occurrence which lately took place in Skye shows its need. Two women, Flora Macbeth, a widow, and her sister, Catherine Macdonald, were seized with typhus fever, and on the neighbours suspecting the nature of their illness the house was boycotted. When the parochial anthorities were informed of the state of affairs, the medical officer visited the house, and found that Catherine had died an hour previous to the time of his visit, and the widow was suffering from typhus fever, although not confined to bed. A nurse was provided, and she remained with the survivor through the night. In the morning her patient, being delirious, got up, and entered an adjoining apartment with a lighted candle, the flame of which caught hold of some dry ferns. The house was soon in a blaze, notwithstanding the efforts of the nurse. She attempted to rescue the widow, but was unsuccessful, and the latter perished, her body being reduced to ashes. The corpse in the coffin was also burnt beyond recognition.

DISTRICT NURSES FOR SURREY.—We have received, in pamphlet form, a reprint of the proceedings of the Benefit Association for Providing Nurses for the Sick which took place at the meeting held on April 20th at Reigate Public Hall. We gave a short account of the meeting at the time, and are glad to hear its ideas have taken practical form. It seems rather a pity that there should be fifteen rules for persons requiring a nurse; we know of many out-of-the-way cottages in the Reigate district where a patient might die ere any of the family could spell out and understand and obey these rules. Take the first rule for instance: "Applications for nurses should be made to a committee lady or to the secretary, accompanied with post card or stamped envelope ready addressed, or if by telegram, with the answer prepaid. No nurse shall attend a case without an order." How different this is to the simple sending a child for nurse which exists in most villages; and then the rules themselves have to be bought for a penny. If it were not that we know the Association is trying to follow Miss Broadwood's successful lead, we should be very doubtful about its future. The charges of the Association are as follow:

| In the Class of | One Order for each Annual Subscription of | Fees for Nurses per Week. To Subscribers | Non-Subscribers. |
|---|---|---|---|
| Labourers ... ... ... | 2*s*. | 1*s*. | 2*s*. |
| Artisans, School Teachers, Gardeners, Gentlemen's Servants living in their own homes, Railway Porters, &c. ... | 3*s*. | 2*s*. | 4*s*. |
| Farmers and Tradespeople ... | 5*s*. | 5*s*. | 10*s*. |
| Gentry ... ... ... | 21*s*. | 15*s*. | 21*s*. |

The fee for Cottage Hospitals and Homes will be 5*s*. per week.

### For Queen and Country.

There was a stir in the great military hospital of the south. The echoes of royalty and its attendant surroundings were only just dying away. The great event to which beating hearts had been looking forward for days had come, and was over. The officials and staff of nurses breathed freely once more, and folded their hands for a brief rest.

The face of one nurse, however, did not relax its anxious lines; she was the youngest, and not yet accustomed to look upon the patients as "cases." Sitting by a bedside in a ward that was strangely still in the gay afternoon sunshine, her eyes were fixed with a tension of watchfulness upon every movement of the young trooper who lay there dying of his wounds. She seemed to be on guard, waiting for some expected change; now and then her soft lips moved, perhaps in prayer.

"Are you still there?" murmured the patient, opening his eyes from a doze. "Ah well, its over and done, and they can't say Jem Ramsay brought discredit on the village, anyway. I've given my life for my Queen and country; and the Queen, God bless her, has pinned the Victoria Cross on my breast; you'll write *that* to the old mother, won't you, nurse? Poor old mither!" and the dying soldier languidly slipped into his native Scotch; "what'll she be doin' to-day? Its Saturday, isn't it? She'll be redding up the old hame (its just a but and a ben), for the morn's the Sabbath, and she'll be away to the kirk with her bit Bible folded in a hankerchief, and a sprig of lad's love on top. I can see her," and his voice grew sharp and clear. "And when they pray for 'our soldiers' she'll say softly: 'Ay, that's our Jem!' I'm all she's got left in this world, nurse, and—soon she'll have nobody. You'll write yourself to her," and the thin fingers twitched the nurse's sleeve.

"Yes, yes, I shall be sure to write, don't fear," was the reply, in a grave, pitying voice.

There was a pause, then the eyes opened again.

"I never saw her before, never; the Queen, I mean. I'm main glad to have seen her before I go. I'd die again for her, I should. She spoke to me, tell mother *that*, too; spoke words like silver sound. And in her eyes there were tears, she was sair sorry for me, ye ken; her eyes minded me of the bits of blue sky that peered down through the treetops in the woods at hame when I, a child, used to lie on the bracken and look up."

The voice grew a little weaker, the Scotch and English mingled more frequently. Then the feeble brain wandered out of the hospital ward, and away off to the plains of Egypt.

"Hold out Carter, I'm coming to ye, mate. Ye black deils, if ye harm a hair of his head I'll ——"

"Hush, hush!" soothed the nurse. Her fingers with a touch of magic strayed over the flushed brow, and the agitated face became calm again.

"Oh, I was only thinking of Carter."

"It was Carter's life you saved, wasn't it?" asked the nurse. "You rode back alone into a group of Arabs who were trying to kill him, and carried him off from them. I heard them telling her Majesty those very words."

"Yes, yes, it was Carter I saved, but he'd have done the same for me, you know," said the trooper simply; then, pulling himself together with an effort, he went on: "Look here, nurse, when you're writing to the old mother, I want you to see that my bits of things are sent to her; and reach down that little box, will you? There's a long string of amber beads I bought in an Algerian bazaar. Will you wear them sometimes to keep you in mind of Jem Ramsay? You've been rare kind to me. And there's the little old Bible I brought from home—it must go to Jeannie. Poor lass! she'd have been my wife; maybe she'll get a better lad some day. Once I couldn't have wished that for her, but now things seem so different. I've lost my hold on life, and the other world is but a stone's throw off. I'm thinking it's never very far from us, only our eyes and our ears are filled with glamour, and we don't see it, nor hear the voices. They're loud enough now, calling, calling. Coming, coming!" he cried out loudly. "It's the great Captain himself! Here!" and there was a silence. Then the soul trembling on the borderland gave one look back to earth: "Listen"—the voice was but a weak whisper—"there's one thing I'd like to take with me. It's this," and he lovingly fingered the Victoria Cross. "You see, I've a fancy to have it on at the great roll-call,' and the words ended with a faint sigh. Another silence, longer, more profound. The nurse was alone, and a little sunbeam came dancing down the wall to kiss the lips now shut for all time. M. B. Manwell.

### The Yellow Fever in Jacksonville.

*(From a Correspondent.)*

Philadelphia, Sept. 11th.

Since the outbreak of yellow fever in Jacksonville, Florida, some three weeks ago, there have been 600 deaths, out of a population of 15,000. It is still raging: yesterday there were 49 new cases, and seven deaths.

Several nurses in this city volunteered their services, but they were declined, as the relief committee decided not to take any doctors or nurses, who had not already had yellow fever.

Miss Clara Barton, President of the Red Cross Association of Nurses at Washington, wired to day to President Mitchell, of the Board of Health, that she would do her best to establish the Red Cross headquarters in Jacksonville.

The following is taken from the *Philadelphia Press*, September 9th:—"The Rev. Mr Sharpe, who died yesterday, was pastor of St. Paul's Church. He had been a resident of this city only a few months, but was well known and deservedly popular with all classes. He had been an indefatigable worker on the Relief Committee, and had nursed and tended the sick in his parish ever since the beginning of the epidemic. He and his whole family were stricken with the disease only four days ago. Of fourteen nurses who reached the city last night from New Orleans, eight were assigned to duty at Sand Hills, the other six to St. Luke's. Eighteen more nurses came in from New Orleans to night, three of them women. One pressing need is thereby filled partially, but fifty more are needed. While the number of new cases to-day may not be large, as soon as the sun shines out with tropical fervour the list will increase to alarming proportions. "Few physicians have volunteered as yet—that is, those who have had experience with yellow fever."

Jacksonville, Sept. 10th.

The official bulletin is as follows: "New cases, 32; deaths, 5. The great lack of nurses here is shown in the case of the Rev. Mr. Sharp, who died lately. He had no one to watch over him save a very ignorant and inexperienced negress. He took a hot bath, and then made her bathe his feet in ice-water and put ice on his neck. She did not know any better, and he died in an hour. Dozens of such instances might be mentioned to prove that many deaths have been caused by lack of good nurses. Last night, when the eighteen nurses from New Orleans arrived there were from three to five applicants for every one; but they had to go to the hospitals, and patients sick at their homes will have to wait until more can come. The Mayor of Charleston telegraphs that a corps of good, experienced nurses is there ready to come at any moment, at Charleston's expense. They will be thankfully accepted to-day, and two hundred more could be used."

The City of New York has collected and sent to the fever-stricken city a sum of 19,286.29 dols., 12,000 dols. of which was contributed by an unknown benefactor.

The City of Philadelphia has also sent a sum of 5,000 dols.

### Lectures.

On October 10th, at St. Thomas's Hospital, at 8 p.m., Dr. Bristowe, F.R.S., in the chair, Mr. E. T. Clifford on the "Position and Prospects of the National Pension Fund for Nurses."

On October 6th, at Hawkstone Hall, Kennington, at 7.30 p.m., the first of a course of lectures by Professor V. B. Lewis, on "Chemistry of our Arts and Manufactures." Fee 5*s*.

On October 9th, at Lecture Hall, Bermondsey, at 8 p.m., the first of a course of lectures by Professor V. B. Lewis, on "Chemistry of Everyday Life." The same course will be delivered at the Town Hall, Walthamstow, commencing October 11th, and at the Town Hall, Stratford, commencing October 10th. All in connexion with the University Extension Movement.

### Why Indeed!

Our contemporary, the *Philanthropist*, publishes the following "Notes by the Man of Ross":—

"The *Nursing Record* is very angry with me because I wrote last month:—

"Mr. Burdett has scored one against the British Nursing Association. When it was proposed that the public should take such an interest in the National Fund for Nurses as to help it with subscriptions, there was a great and indignant cry against a suggestion so humiliating to independent people like nurses. Their leaders were very lofty in their wrath. They characterised the suggestion as 'insulting.' But now the Princess Christian suggests that the British Nursing Association is in need of public subscriptions to carry out their laudable scheme. And Mr. Burdett wants to know why. Is registration such a costly process? Or is the present registration scheme too ambitious? That the public should subscribe to help a pension fund for nurses one understands; but what funds does a scheme of registration require?"

"Whereupon the writer of 'Nursing Echoes' in the *Record* grows amusing in the following deliciously and femininely spiteful paragraph:

"Now it is, of course, not necessary to tell my readers that the context shows the writer is referring to the British Nurses' Association, and that his use of the term *Nursing* Association simply proves that he knows so little of the subject as to think that nurses and nursing are synonymous terms! Then he infers that the leaders of the Association made 'a great and indignant cry' against the Pension Fund. I inquired carefully into this matter last week, and can now assert that this is perfectly, totally untrue. So the Editor of *The Philanthropist* either knows nothing at all of the subject, and so made this misstatement in pure ignorance, or else he has wilfully and deliberately published it. knowing that it was untrue. I am told, on the best authority, that the *leaders of the Association* have never exhibited the slightest wrath, 'lofty' or otherwise, or cried greatly and indignantly or otherwise, or taken the slightest official notice of any kind of, or against, the Pension Fund."

"Notice the positive alternative of wilfully and deliberately publishing what is untrue. It is either ignorance or falsehood—the dagger or the bowl, and all because of an 'inference.' Queen Eleanor herself was hardly more fierce. But, I ask any reader whether my words 'their leaders' do not plainly and grammatically refer to the 'leaders' of 'nurses.' Of course, like everyone else, I share the common belief that the promoters of the 'British Nurses' Association' and of the *Nursing Record*, which is full of their announcements, are identical. And why should they not be?

"But I am offered the choice of 'ignorance' or 'falsehood'—the dagger or the bowl. People not so good as nurses, not so wholly devoted, I mean, to the spirit of charity as nurses are, might have suggested 'error,' 'natural inferiority of man,' and such like civilized terms. No! it is the dagger or the bowl—ignorance or falsehood. For my part I plead ignorance, not *that other*—no, ignorance, *if you please*. I did not know that the writers of the *Nursing Record* were *not* leaders of the nurses, and I will not again make the mistake of assuming that they are.

"And all because Mr. Burdett 'scored one.' I can only add that Mr. Burdett will score a great many more than one if the *Nursing Record* continues to write in its present hysterical style. Why does it not show—that good representative of the good—some of the charity, self-control, and kindliness which it is always preaching about to nurses."

### Appointments.

[It is requested that successful candidates will send a copy of their applications and testimonials, with date of election, to The Editor, The Lodge, Porchester Square, W.]

Jamaica Lunatic Asylum.—We are glad to be able to announce that Miss A. E. Blake has secured the lucrative post of matron to this asylum. Miss Blake trained at the Birmingham Institution for Nurses, where she remained four years, then for six years she had the supervision of the female side of Parkside Asylum, Macclesfield, containing 344 beds. For the last three years Miss Blake has worked for the St. Pancras Board of Guardians, first as superintendent of nurses at their school infirmary, and latterly in the new wing of the Workhouse, which contains 486 beds. Miss Blake's certificates and testimonials are of such an unusually high order, we are not surprised she obtained the appointment. Many of our readers must remember the charming anecdotes signed "A. E. B." which have appeared in our pages, and are faithful records of some of Miss Blake's nursing adventures. All who enjoyed those amusing stories will look forward to hearing of Miss Blake's further experiences when she reaches "fresh fields and pastures new."

### Vacancies.

The following vacancies are announced:—

**Matrons.**

Sheffield General Infirmary; £70
Tunbridge Wells Eye Hospital; £25

**Nurses.**

Kingston Convalescent Home; £25

Homes for Aged Mariners; £25.

Chester Deaconess' Institution; £30

Victoria Park Chest Hospital; £24

Ipswich Nurses Home; £22; three.

Kent Nursing Institution; several.

Tunbridge Wells General Hospital; £25.

Merthyr General Hospital; £25

St. Saviour's Infirmary; £20; several.

Royal Hospital for Children and Women, Waterloo Bridge Road.

Tonbridge Union; £35.

Nurses' Home. Truro; two.

Stockton Surgical Hospital; £20.

Canterbury Institute; two.

Royal Alexandra Children's Hospital; £20.

St. Saviour's Infirmary, East Dulwich Grove; Several.

Beckett Hospital Barnsley; £25.

Mr. D. E. Wilson, of 96, 97, 98*a* Wimpole Street, London, W., has several vacancies for Male and Female Nurses.

**District Nurses.**

Disley; £70.
Exmouth; £30

**Probationers.**

Hospital for Consumption, Ventnor.

Lloyd Cottage Hospital.

Buckinghamshire General Infirmary.

Royal Hospital for Diseases of the Chest.

Lancaster Infirmary; £13.

"The Hospital" as a Medium for Advertisers.—We have received the following letter from the Matron of Buckinghamshire General Infirmary: "I have received so many applications from the advertisement you inserted for me that I beg to ask if you would please enter the following, instead of the advertisement again. 'Probationer engaged? Please accept this in answer to the numerous applications of last week received by the Matron,' and oblige yours truly, S. Middleton (Matron)."

### Notes and Queries.

To Correspondents.—1. Questions may be written on post-cards. 2. Advertisements in disguise are inadmissible. 3. In answering a query please quote the number. 4. If a private answer is desired, a stamped addressed envelope must be forwarded.

**Queries.**

(66). *Matrons and Sisters in Foreign Hospitals.*—Nurse Rachel writes: "Can you, or any of your readers, kindly tell me the best way of obtaining information respecting the vacant posts in the hospitals abroad, for matrons, sisters, or head nurses?

(67) *Medical Terms and Prescriptions.*—Will you kindly inform me what book would best enable me to learn the medical (Latin) names of diseases, and to understand physician's prescriptions.—E. G.

(68) Can anyone tell a lady, certificated nurse and masseuse, who has had great experience in the management of similar institutions, of a good opening abroad for a medical and nurses' home? Distance from England or objection. Address "Massage," Office of The Hospital.

**Answers.**

(67) *E. G.* will find "Hoblyn's Dictionary of Medical Terms" (Whittaker and Co.) useful. The first four chapters in "Lessons or Precriptions," by W. Handscel Griffiths (Macmillan and Co.), may also be found helpful.

(66). *Nurse C.* can get the work of Dr Allan, on "Infectious Fevers," from Messrs. J. and A. Churchill, 11, New Burlington Street.

We cannot answer *N.P.'s* first question; we should advise her to seek medical aid if her shoulders are very bad. With regard to works on nursing, they are very numerous; but perhaps "Domville's Manual," would prove most useful. Price 2*s.* 6*d.*, from Messrs. Churchill.

